# A linked independent component analysis framework for characterizing site-effect patterns in multi-site structural and functional MRI

**DOI:** 10.3389/fbinf.2026.1902380

**Published:** 2026-07-29

**Authors:** Huashuai Xu, Yuge Xing, Weiya Guo

**Affiliations:** 1 Women and Children’s Hospital of Dalian University of Technology, Dalian, China; 2 School of Biomedical Engineering, Faculty of Medicine, Dalian University of Technology, Dalian, China; 3 Department of Pediatrics, Linyi People’s Hospital, Linyi, China

**Keywords:** acquisition parameters, inter-site variability, linked independent component analysis, multi-site MRI, resting-state fMRI, site effects, structural MRI

## Abstract

**Introduction:**

Large-scale multi-site magnetic resonance imaging (MRI) improves population coverage and statistical power, but scanner- and protocol-related variability can obscure biological effects. Most harmonization methods aim to reduce site-related variance for downstream analysis, whereas less attention has been paid to where site effects are spatially expressed, whether they are reproducible across site compositions, and which acquisition parameters contribute to them.

**Methods:**

We developed a modality-wise Linked Independent Component Analysis (LICA) framework to identify and interpret site-effect patterns in structural and resting-state functional MRI. Grey matter (GM) volume, amplitude of low-frequency fluctuation (ALFF), and regional homogeneity (ReHo) maps were analyzed separately. For each imaging measure, LICA decomposed voxel-wise maps into spatial components and subject-level loadings. Components were classified according to their associations with site labels and biological covariates, their spatial reproducibility was assessed using stepwise site-inclusion analyses, and their technical attribution was evaluated using cross-validated models based on site labels and recorded acquisition parameters. The framework was applied to ABIDE II GM maps from 913 participants across 18 sites and ALFF and ReHo maps from 795 participants across 16 sites.

**Results:**

LICA identified site-related components across all three imaging measures. Site effects were not limited to uniform global shifts, but formed modality-specific spatial patterns. GM volume showed a dominant and highly stable whole-brain site-effect pattern, together with site-specific and regional components. In contrast, ALFF and ReHo showed more heterogeneous functional patterns, including global, focal, and scattered configurations. Site labels explained the largest proportion of loading variance, whereas recorded acquisition parameters showed modality-dependent contributions: TR and TE were more prominent for structural site effects, while FA, voxel size, TR, and scanner model contributed more strongly to functional site effects.

**Discussion:**

The proposed framework provides a component-level diagnostic approach for multi-site MRI analysis. By mapping, stabilizing, and technically interpreting site-effect patterns, it complements conventional harmonization methods and may improve the transparency and reproducibility of multi-site structural and functional MRI studies.

## 1 Introduction

Magnetic resonance imaging (MRI) has become a central tool for investigating human brain structure and function *in vivo*, providing non-invasive measurements of brain morphology, intrinsic functional activity, and inter-individual variability. Over the past 2 decades, neuroimaging research has increasingly moved from single-site studies toward large-scale, multi-site data collection, driven by the need for larger samples, broader demographic and clinical coverage, and more reproducible statistical inference (Biswal et al., 2010; Di Martino et al., 2017; Nichols et al., 2017; Zuo et al., 2014). In parallel, the field has also shifted from single-modality analysis toward multimodal characterization of the brain (Calhoun and Sui, 2016; Sui et al., 2012a; Sui et al., 2023; Sui et al., 2012b). Structural MRI provides information about brain morphology, whereas resting-state functional MRI captures spontaneous neural activity and local functional organization through measures such as the amplitude of low-frequency fluctuation (ALFF) and regional homogeneity (ReHo) (Zang et al., 2004; Zang et al., 2007). Integrating structural and functional measures across multiple sites therefore provides an opportunity to characterize brain variability more comprehensively than any single-site study.

However, the scientific advantages of multi-site MRI are accompanied by substantial technical heterogeneity. MRI-derived measurements are influenced by scanner manufacturer and model, head coil, software version, pulse-sequence implementation, reconstruction procedure, and acquisition parameters such as repetition time (TR), echo time (TE), flip angle, and voxel size (Chen et al., 2014; Fortin et al., 2017; 2018; Hu et al., 2023). These factors can introduce systematic site-related variability into both structural and functional MRI data. In structural MRI, site effects may bias estimates of grey matter volume, cortical thickness, or other morphometric features (Fortin et al., 2018; Maikusa et al., 2021). In resting-state functional MRI, differences in acquisition and measurement conditions can affect temporal fluctuation amplitudes, local synchronization measures, and functional connectivity estimates (Wang et al., 2023; Yamashita et al., 2019; Yu et al., 2018). If not appropriately addressed, such site-related variability can obscure true biological effects, inflate false-positive findings, and reduce reproducibility across studies. Consequently, a wide range of statistical and machine-learning harmonization methods have been developed to mitigate site effects in multi-site neuroimaging data (An et al., 2022; Fortin et al., 2017; Gardner et al., 2025; Hao et al., 2023; 2026; Ho et al., 2026; Lu et al., 2025; Moazami et al., 2026; Nugent et al., 2026; Xiao et al., 2026; Xin et al., 2026; Xu et al., 2023a; Zuo et al., 2023; 2026). Together, these methods have substantially improved the feasibility of pooled analyses across heterogeneous imaging sites.

Despite these advances, most harmonization methods are primarily designed to reduce site-related variability for downstream pooled inference, rather than to characterize the site effects themselves. Statistical approaches such as ComBat and general linear model (GLM)-based adjustment typically model site as a nuisance factor and rely on assumptions about the relationship between technical and biological variables. When site is partially confounded with diagnosis, age, sex, or other biological covariates, aggressive adjustment may attenuate meaningful biological effects or introduce residual bias (Fortin et al., 2017, 2018; Johnson et al., 2007). Deep-learning approaches can provide flexible nonlinear harmonization, but their learned transformations are often difficult to interpret, and distributional alignment alone does not necessarily indicate which brain regions, imaging features, or acquisition parameters drive inter-site variability (An et al., 2022; Dinsdale et al., 2021; Hu et al., 2023; Wang et al., 2023). Similarly, although independent component analysis (ICA)-based methods can separate structured components related to scanner or site effects, they have more often been used as denoising or correction tools than as a systematic framework for identifying reproducible site-effect patterns and linking them to concrete technical factors (Chen et al., 2014; Feis et al., 2015; Li et al., 2020). Therefore, a critical gap remains: site effects are still poorly characterized as spatially organized, reproducible, and technically interpretable imaging patterns. Without such characterization, it is difficult to determine whether a site-related component reflects a stable scanner–protocol effect, a site-specific outlier, or variance partially entangled with biological covariates. Mapping the spatial distribution, cross-site stability, and acquisition-parameter explainability of site effects is therefore essential for moving multi-site MRI analysis from empirical variance removal toward interpretable diagnosis of measurement heterogeneity.

Although site effects have been widely recognized in multi-site MRI, most studies have treated them primarily as nuisance variability to be removed rather than as technical patterns to be analyzed. A small number of studies have begun to examine the sources of MRI measurement variability more directly. For example, a recent phantom-based radiomics study (Hajianfar et al., 2024) systematically varied scanner type, inversion recovery, flip angle, and repeated scans while controlling other acquisition conditions, and showed that scanner differences exerted the strongest influence on MRI radiomic features, followed by inversion recovery and flip angle, whereas repeated scans on the same scanner had the smallest effect. This type of controlled design demonstrates that site-related variability can be decomposed into contributions from specific technical factors. However, phantom data may not fully reflect real-world multi-site human MRI acquisition, where scanner-protocol differences, participant characteristics, motion, and preprocessing effects are jointly present. Moreover, these studies mainly evaluated the robustness of extracted radiomic features and did not investigate the spatial distribution of site effects in brain MRI.

In this study, we propose a modality-wise neuroimaging framework for identifying, characterizing, and interpreting stable site-effect patterns in multi-site MRI data using Linked Independent Component Analysis (LICA). Rather than treating site effects only as nuisance variance to be removed, the proposed framework uses LICA to decompose structural and resting-state functional MRI features into spatially distributed components with corresponding subject-level loadings. These loadings are then examined in relation to site labels, biological covariates, and acquisition parameters. Specifically, the framework was designed to address three related questions. First, in each imaging modality, component-wise association analysis was used to identify site-related components and distinguish site-dominant components from mixed components that were also associated with biological variation. Second, stepwise site-inclusion analysis was used to determine which site-related components were spatially stable and to characterize their site-effect distribution patterns. Third, site labels and recorded acquisition parameters were used to quantify the contribution of site-related variables to component loadings, thereby interpreting the weights of the identified site-effect patterns. By applying this framework to structural MRI and resting-state functional MRI data from the Autism Brain Imaging Data Exchange II dataset, we aimed to determine whether site effects exhibit reproducible spatial organization across modalities and to what extent these patterns can be attributed to measurable technical factors.

## 2 Materials and methods

### 2.1 Dataset

We used data from the publicly available Autism Brain Imaging Data Exchange II (ABIDE II) repository (http://fcon_1000.projects.nitrc.org/indi/abide/abide_II.html) to evaluate the proposed framework. ABIDE II is a large-scale, multi-site neuroimaging initiative designed to facilitate studies of autism spectrum disorder (ASD) and typical development by aggregating structural and functional MRI data acquired across independent imaging sites (Di Martino et al., 2014, 2017). The present study included both structural MRI (sMRI) and resting-state functional MRI (rs-fMRI) data. Participants were retained if they had usable imaging data, available demographic information, diagnostic labels, and sufficient acquisition metadata for site-effect analysis. Participants with missing key covariates or visible imaging artifacts were excluded during quality control.

For the structural MRI analysis, high-resolution T1-weighted images were included from 913 participants across 18 sites, comprising 402 individuals with ASD and 511 healthy controls (HC). These data were acquired on scanners from multiple manufacturers, including Siemens, Philips, and GE, with substantial variation in scanner models and acquisition protocols. The recorded acquisition parameters included scanner manufacturer and model, repetition time (TR), echo time (TE), flip angle (FA), and voxel size. These parameters were used in subsequent analyses to quantify the extent to which recorded acquisition settings explained the loadings of site-related components. The site-specific structural MRI acquisition parameters are summarized in Table 1, and the corresponding demographic information, including diagnostic group, age, and sex distribution, is summarized in Table 2.

**TABLE 1:** Scanning parameters for structural MRI data from ABIDE II.

Sites	Scanners	TR/TE (ms)	FA (degree)	Voxel size
EMC	GE MR750	10.27/4.24	16	0.9 × 0.9 × 0.9
ETH	Philips Achieva	8.4/s	8	0.9 × 0.9 × 0.9
GU	Siemens TriTim	2,530/3.5	7	1 × 1 × 1
IU	Siemens TriTim	2,400/2.3	8	0.7 × 0.7 × 0.7
KKI	Philips Achieva	8/3.7	8	1 × 1 × 1
KUL	Philips Achieva	9.4/4.6	8	1.2 × 1.2 × 1.2
OHSU	Siemens TriTim	2,300/3.58	10	1 × 1 × 1.1
ONRC	Siemens Skyra	2,200/2.88	13	0.8 × 0.8 × 0.8
SU	GE SIGNA	5.9/1.8	11	0.9 × 0.9 × 1
UCD	Siemens TriTim	2000/3.16	8	1 × 1 × 1
UCLA	Siemens TriTim	2,300/2.86	9	1 × 1 × 1.2
UM	GE Healthcare	−/−	12	1 × 1 × 1
USM	Siemens TriTim	900/2.91	9	1 × 1 × 1.2
BNI	Philips Ingenia	s/s	9	1.1 × 1.1 × 1.2
IP	Philips Achieva	25/5.6	30	1 × 1 × 1
NYU	Siemens Allegra	3.25/3.25	7	1.3 × 1.0 × 1.3
SDSU	GE MR750	8.14/3.17	8	1 × 1 × 1
TCD	Philips Achieva	8.4/3.9	8	0.9 × 0.9 × 0.9

s, shortest.

Data were collected from 18 different sites: Erasmus University Medical Center (EMC), ETH Zürich (ETH), Georgetown University (GU), Indiana University (IU), Kennedy Krieger Institute (KKI), Katholieke University Leuven (KUL), Oregon Health and Science University (OHSU), Olin Neuropsychiatry Research Center (ONRC), Stanford University (SU), University of California Davis (UCD), University of California Los Angeles (UCLA), University of Miami (UM), University of Utah School of Medicine (USM), Barrow Neurological Institute (BNI), Institut Pasteur and Robert Debré Hospital (IP), NYU Langone Medical Center (NYU), San Diego State University (SDSU), and Trinity Center for Health Sciences (TCD).

**TABLE 2:** Demographic information of structural MRI data from ABIDE II.

Sites	ASD/HC	Age	Sex (male/female)
EMC	18/20	8.28 ± 1.12	31/7
ETH	8/17	22.43 ± 4.19	25/0
GU	33/43	10.74 ± 1.64	51/25
IU	18/18	24.61 ± 7.70	28/8
KKI	32/133	10.36 ± 1.30	103/62
KUL	7/0	21.71 ± 3.04	7/0
OHSU	33/51	10.94 ± 2.02	52/32
ONRC	16/29	23.24 ± 3.91	32/13
SU	15/17	10.99 ± 1.15	29/3
UCD	13/13	10.04 ± 1.77	19/7
UCLA	12/12	11.04 ± 2.46	19/5
UM	7/12	10.32 ± 2.06	14/5
USM	13/16	21.21 ± 8.26	24/5
BNI	29/26	37.85 ± 15.59	55/0
IP	22/31	20.65 ± 10.36	25/28
NYU	75/29	9.05 ± 4.50	94/10
SDSU	32/25	13.1 ± 3.10	48/9
TCD	19/19	15.40 ± 3.18	38/0
Total	402/511	14.98 ± 9.42	694/219

For the resting-state functional MRI analysis, rs-fMRI data were included from 795 participants across 16 sites. Functional MRI data were analyzed using two commonly used voxel-wise measures of spontaneous brain activity: amplitude of low-frequency fluctuation (ALFF), which reflects the intensity of low-frequency temporal fluctuations, and regional homogeneity (ReHo), which reflects local synchronization of the resting-state BOLD signal. The acquisition parameters were retained for parameter-explainability analyses of functional site-related components. The site-specific rs-fMRI acquisition parameters are summarized in Table 3, and the corresponding demographic characteristics are summarized in Table 4.

**TABLE 3:** Scanning parameters for functional MRI data from ABIDE II.

Sites	Scanners	TR/TE (ms)	FA (degree)	Voxel size
EMC	GE MR750	2,000/30	85	3.6 × 3.6 × 4.0
ETH	Philips Achieva	2000/25	90	3 × 3 × 3
GU	Siemens TriTim	2,000/30	90	3 × 3 × 3
IU	Siemens TriTim	813/28	60	3.4 × 3.4 × 3.4
KKI	Philips Achieva	2,500/30	75	3 × 3 × 3
KUL	Philips Achieva	2,500/30	90	1.6 × 1.6 × 3.1
OHSU	Siemens TriTim	475/30	60	3 × 3 × 3
ONRC	Siemens Skyra	2,500/30	90	3.8 × 3.8 × 3.8
SU	GE SIGNA	2,000/30	80	3.4 × 3.4 × 3.5
UCD	Siemens TriTim	2,000/24	90	3.5 × 3.5 × 3.5
UCLA	Siemens TriTim	3,000/28	90	3 × 3 × 4
UM	GE Healthcare	2,000/30	75	3.4 × 3.4 × 3.4
USM	Siemens TriTim	2,000/28	90	3.1 × 3.1 × 4
BNI	Philips Ingenia	3,000/25	80	3.8 × 3.8 × 4
IP	Philips Achieva	2,700/45	90	3.6 × 3.7 × 4
NYU	Siemens Allegra	2,000/15	90	3 × 3 × 4
SDSU	GE MR750	2,000/30	90	3.4 × 3.4 × 3.4
TCD	Philips Achieva	2,000/27	90	3 × 3 × 3.2

**TABLE 4:** Demographic information of fMRI data from ABIDE II.

Sites	ASD/HC	Age	Sex (male/female)
EMC	14/13	8.39 ± 1.03	22/5
ETH	7/22	23.36 ± 4.59	29/0
GU	27/41	10.89 ± 1.62	46/22
IU	18/19	24.62 ± 7.59	28/9
KKI	25/123	10.37 ± 1.27	89/59
KUL	25/0	23.76 ± 5.10	25/0
OHSU	33/51	11.00 ± 2.04	52/32
ONRC	15/26	23.24 ± 4.09	30/11
SU	14/17	10.94 ± 1.14	28/3
UCD	—	—	—
UCLA	12/12	11.04 ± 2.46	19/5
UM	—	—	—
USM	9/12	24.34 ± 7.49	17/4
BNI	29/28	38.86 ± 15.41	57/0
IP	13/21	22.37 ± 10.97	16/18
NYU	61/28	9.24 ± 4.78	81/8
SDSU	30/24	13.19 ± 3.06	46/8
TCD	9/17	15.98 ± 3.23	26/0
Total	341/454	15.85 ± 10.20	611/184

In this study, “site” refers to the acquisition site and its associated scanner–protocol configuration, rather than to a purely geographical location. Site effects were treated as technical variability arising from differences in scanners, acquisition protocols, and other site-specific measurement conditions. In contrast, diagnosis, age, and sex were treated as biological or participant-level variables. This distinction was used throughout the analysis to classify LICA components as site-dominant components or components jointly associated with site and biological variables.

### 2.2 MRI preprocessing and derivation of features

High-resolution T1-weighted structural MRI data were processed to generate grey matter (GM) volume maps using FSL-VBM (https://fsl.fmrib.ox.ac.uk/fsl/fslwiki/FSLVBM). First, non-brain tissues were removed from the native T1-weighted images, and the brain-extracted images were segmented into GM, white matter (WM), and cerebrospinal fluid (CSF). The GM images were affine-registered to the MNI152 standard space, concatenated, and averaged to create an initial study-specific GM template. To reduce hemispheric bias, the averaged GM image was mirrored along the left-right axis, and the original and mirrored images were re-averaged to generate a symmetric affine template. All GM images were then non-linearly registered to this initial template, concatenated into a four-dimensional image, and averaged to construct the final symmetric, study-specific non-linear GM template in MNI152 space with a spatial resolution of 2 × 2 × 2 mm^3^. This template was generated using all included participants to avoid preferential registration toward any diagnostic group or acquisition site. Native-space GM images were subsequently non-linearly registered to the final template and modulated to preserve local volume information. The modulated GM maps were smoothed using an isotropic Gaussian kernel with a sigma of 3 mm. The final GM volume maps, resampled to 2 × 2 × 2 mm^3^, were used as the structural MRI features for subsequent analysis.

Resting-state functional MRI data were preprocessed using FSL FEAT following a standardized pipeline. For each participant, the first six volumes were discarded to reduce the influence of magnetization instability. Motion correction was performed using the Motion Correction Linear Registration Tool (MCFLIRT), with all functional volumes aligned to the middle volume of the time series. The motion-corrected functional images were then spatially normalized to the MNI152 standard space and resampled to 2 × 2 × 2 mm^3^. These preprocessed resting-state fMRI data were used to derive voxel-wise functional measures of spontaneous brain activity.

Two resting-state functional MRI measures, amplitude of low-frequency fluctuation (ALFF) and regional homogeneity (ReHo), were derived from the preprocessed fMRI data using DPABI (Yan et al., 2016). ALFF quantifies the power of spontaneous low-frequency BOLD signal fluctuations within the 0.01–0.1 Hz frequency range and reflects regional intrinsic activity. ReHo measures local synchronization of resting-state BOLD time series by estimating the concordance between a voxel and its neighboring voxels, thereby reflecting regional functional coherence. These two measures were selected because they capture complementary aspects of spontaneous brain activity: ALFF emphasizes fluctuation amplitude, whereas ReHo emphasizes local temporal coherence. For ALFF, spatial smoothing was performed before calculating low-frequency fluctuation amplitudes (Yang et al., 2020). For ReHo, spatial smoothing was applied after ReHo estimation using a Gaussian kernel with a full width at half maximum (FWHM) of 6 mm to preserve local synchronization patterns during calculation (Jia et al., 2019). The resulting GM volume, ALFF, and ReHo maps were all normalized to the same 2 × 2 × 2 mm^3^ MNI152 space and used as imaging measures in the subsequent LICA-based site-effect analysis.

### 2.3 LICA-based identification and characterization of modality-specific site-effect patterns

Among data-driven decomposition techniques, we selected Linked Independent Component Analysis (LICA) for its methodological robustness and suitability for multi-site neuroimaging. LICA extends standard ICA with a hierarchical Bayesian formulation that jointly models multiple datasets or modalities while preserving statistical independence of the recovered components (Groves et al., 2011). The Bayesian estimation—and associated parameter updates—yields highly stable solutions, such that repeated runs with identical input produce identical or near-identical components, reducing variability due to random initialization that is common in conventional ICA. In practice, LICA balances contributions across datasets, down-weights noise-dominated structure, and retains components that are both independent and informative. This combination of stability and interpretability makes LICA well-suited to identify reproducible site-effect patterns in multi-site MRI, where consistent spatial detection across analyses is critical. LICA has been extensively used in neuroimaging studies to reveal biologically relevant components (Doan et al., 2017a; Doan et al., 2017b; Duda et al., 2025; Groves et al., 2012; Li et al., 2020; Liu et al., 2022; Maglanoc et al., 2020; Rokicki et al., 2021; Sui and Calhoun, 2025).

Although LICA has been developed and widely used for multimodal data fusion, the present study did not perform joint multimodal fusion of structural and functional measures. Instead, GM volume, ALFF, and ReHo maps were analyzed separately, allowing site-effect patterns to be characterized in a modality-specific manner. This design was chosen because the primary aim of the present study was not to improve site-effect detection through multimodal fusion, but to examine how site effects are spatially expressed, how stable these patterns are across different site compositions, and how strongly they can be explained by recorded acquisition parameters within each imaging modality. Previous studies have shown that multimodal fusion can improve the identification of site-related variance, but separate modality-wise analyses are more suitable here for comparing the spatial organization and technical origins of site effects across structural and functional MRI measures.

For each imaging measure, LICA was performed separately on the corresponding preprocessed voxel-wise maps. Before decomposition, each modality was organized into a voxel-by-subject data matrix, denoted as Y∈**R** ^V×N^, where Y indicates the imaging measure, Vis the number of voxels, and N is the number of participants available for that modality. For structural MRI, N = 913; for ALFF and ReHo, N = 795. Within each modality, the data matrix was modeled as a linear combination of spatially independent components and their corresponding subject-level loadings, as shown in Equation 1:

Y=XH+E (1)

where X∈**R** ^V×K^ contains the spatial independent components, H∈**R** ^K×N^ contains the subject coefficients (loadings) representing each subject’s contribution to this component, K is the number of components, and E is residual noise. The initial number of components K was set conservatively high so that the Bayesian procedure could down-weight and effectively prune noise-dominated structure (Groves et al., 2011, 2012; Xu et al., 2023b). Concretely, we preset K by sample size: K = 50 for N < 400, K = 100 for 400 ≤ N ≤ 800, and K = 150 for N > 800. This policy is motivated by the fact that LICA can effectively determine the optimal effective dimensionality by down-weighting and gradually eliminating weak components; thus, we preset a sufficiently large K and allow the model to prune negligible components during estimation. This conservative initialization was used to avoid under-specifying the decomposition space. In our previous work (Xu et al., 2023b), we showed that the preset component number can affect linked component formation in multimodal LICA, particularly because lower model dimensionality may force modality-specific sources of variance to be represented within shared linked components. In the present study, however, GM volume, ALFF, and ReHo were analyzed separately rather than jointly fused. Therefore, the component number was not treated as the main experimental variable; instead, a sufficiently high initial dimensionality was used to allow the Bayesian procedure to suppress weak or noise-dominated components while preserving interpretable site-related spatial patterns.

For each component k, we quantified associations between the loading vector H and all covariates. Site association was assessed using one-way analysis of variance (ANOVA) across all site labels. Associations with categorical biological variables, including diagnosis and sex, were assessed using group-wise statistical tests, whereas associations with age were assessed using Pearson correlation. All p-values from the component-wise association analyses were corrected for multiple comparisons using the Bonferroni method. Specifically, Bonferroni correction was applied across the tested components and covariates within each imaging measure, and statistical significance was determined using a family-wise error (FWE) threshold of p < 0.05. A component was defined as a site-dominant component if its loadings were significantly associated with site but not with any measured biological covariate. A component was defined as a mixed component if its loadings were significantly associated with both site and at least one biological covariate. For mixed components, the effects of biological variables were regressed out before the acquisition-parameter explainability analysis. Specifically, diagnosis, age, and sex were removed from the component loadings using a linear regression model, and the residualized loadings were then used to assess their associations with scanner and acquisition parameters. This step was used to reduce the direct influence of measured biological variables when interpreting the technical sources of mixed components. This classification was performed separately for GM volume, ALFF, and ReHo, allowing site-related components to be identified within each imaging measure.

To evaluate the spatial reproducibility of site-effect patterns, we repeated the LICA analysis within each imaging measure using subsets with progressively increasing numbers of sites. For structural MRI, LICA was repeated from 2 up to all 18 sites; for resting-state functional MRI measures, LICA was repeated from 2 up to all 16 sites. In each modality-wise analysis, the full-dataset solution was defined as the reference LICA decomposition obtained using all available sites for that modality, namely, 18 sites for GM volume and 16 sites for ALFF and ReHo. Components estimated from each site-subset run were then matched to the corresponding reference components by maximizing the spatial Pearson correlation between component maps. A component was considered spatially stable if a corresponding site-effect pattern was consistently detected across consecutive site-subset analyses and showed persistently high spatial similarity to the full-dataset reference map. This procedure allowed us to identify site-related components whose site-effect patterns were not dependent on a single site composition, but instead reflected reproducible spatial patterns within each imaging measure.

We then quantified how strongly stable site-effect components relate to acquisition parameters using leave-one-out cross-validation (LOOCV) (Stone, 1974) at the subject level. For each stable component, we modeled subject loadings as a function of acquisition settings—repetition time (TR), echo time (TE), flip angle (FA), voxel size, scanner manufacturer, and scanner model—with age, sex, and diagnostic group included as covariates. In addition to these parameter-specific models, we fitted a site-label model to quantify the overall site-level explainability of each component loading. The predictive performance of the site-label model was then compared with that of models based on individual recorded acquisition parameters. At each LOOCV iteration, one subject was held out, the model was fitted on the remaining N−1 subjects, and the left-out loading was predicted. Aggregating across iterations provided cross-validated performance indices (e.g., $R_{\text{LOO}}^{2}$ , mean absolute error) that summarize the predictive linkage between component loadings and acquisition parameters. To facilitate interpretation, subjects were additionally grouped by parameter levels (e.g., TR/TE bins, FA and voxel-size levels, manufacturer and model categories), and partial correlations between loadings and parameter levels were computed while controlling for the same covariates.

The predictive performance was summarized by the cross-validated coefficient of determination $R_{\text{LOO}}^{2}$ , as defined in Equation 2:

$$R_{\text{LOO}}^{2}=1\;‐\;\frac{\sum_{\text{i}=1}^{\text{N}}{\left(y^{i}‐\hat{y^{i}}\right)}^{2}}{\sum_{\text{i}=1}^{\text{N}}{\left(y^{i}‐\bar{y}\right)}^{2}}$$ (2)

where $y^{i}$ is the observed loading of subject i, $\hat{y^{i}}$ is the predicted loading from the model trained without subject i, and $\bar{y}$ is the mean of all observed loadings. The value of $R_{\text{LOO}}^{2}$ can be negative when the prediction error exceeds that of the mean-loading model, whereas values closer to 1 indicate better predictive performance and a larger proportion of loading variance explained under cross-validation. This analysis was performed separately for GM volume, ALFF, and ReHo, allowing the technical attribution of site-effect patterns to be compared across structural and functional MRI measures.

**FIGURE 1:**
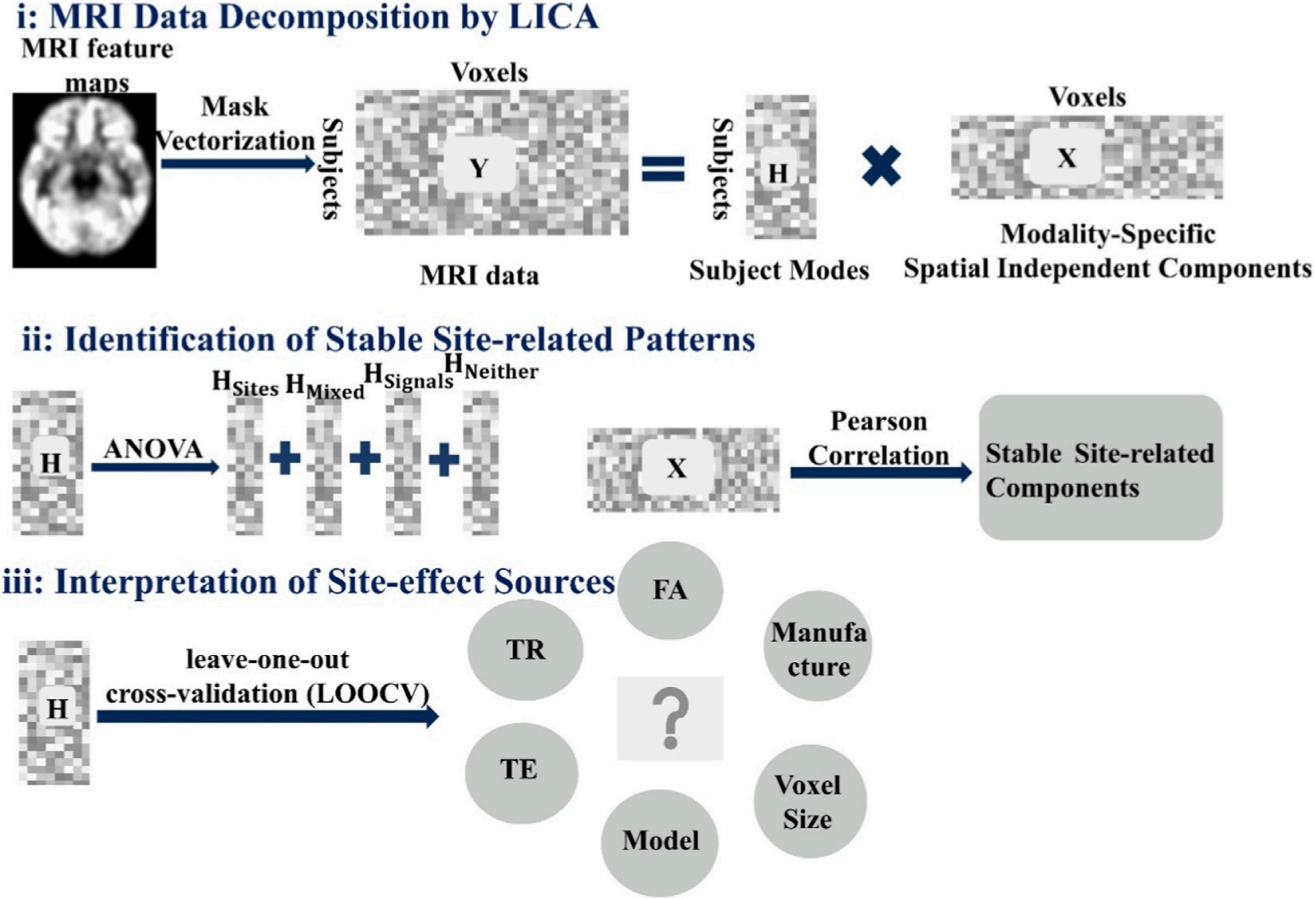
Workflow of the LICA framework: preprocessing, classification, stability, and explainability.

Overall, the proposed analysis was performed independently for GM volume, ALFF, and ReHo. For each imaging measure, the workflow consisted of four steps: LICA decomposition, association-based component classification, spatial stability assessment across site-subset analyses, and acquisition-parameter explainability analysis using $R_{\text{LOO}}^{2}$ . This modality-wise strategy allowed us to compare the spatial organization, reproducibility, and technical attribution of site-effect patterns across structural and resting-state functional MRI measures without performing joint multimodal fusion. A schematic overview of the full analytical workflow is shown in Figure 1.

All LICA analyses and *post hoc* statistical analyses were performed in MATLAB R2023a (https://uk.mathworks.com/). LICA was implemented using FMRIB’s Linked Independent Component Analysis (FLICA) toolbox (https://fsl.fmrib.ox.ac.uk/fsl/fslwiki/FLICA), which was installed separately following the official FSL/FLICA instructions. The same implementation was used for the full-dataset decomposition and all stepwise site-inclusion analyses.

## 3 Results

Across GM volume, ALFF, and ReHo, the LICA-based framework identified site-related components with distinct spatial configurations, stability profiles, and acquisition-parameter associations. The Results section is organized by imaging measure. For each measure, we summarize the number and type of site-related components and then focus on representative patterns, emphasizing their spatial extent, stability across site compositions, site-wise loading distribution, and acquisition-parameter explainability.

### 3.1 Site-effect patterns in GM volume

For the structural MRI analysis, GM volume maps from 913 participants across 18 sites were analyzed using the modality-wise LICA framework. The structural MRI data showed substantial inter-site variation in scanner and acquisition settings, including scanner manufacturer and model, repetition time (TR), echo time (TE), flip angle (FA), and voxel size. In the full 18-site structural MRI dataset, LICA identified 114 independent components, of which 67 were significantly associated with site. Among these site-related components, 63 were classified as site-dominant components and 4 as mixed components associated with both site and at least one biological covariate (see Table 5). This predominance of site-dominant components suggests that structural GM maps contained strong scanner- or protocol-related variability that was not directly explained by the measured biological covariates. The number of site-related components increased progressively as more sites were included, suggesting that additional scanner–protocol heterogeneity introduced new or stronger site-effect patterns in the GM volume data.

**TABLE 5:** Results from LICA decomposition for GM volume.

Data	Subjects	Components	Site-related components
2 sites	63	6	1 (0 + 1)
3 sites	139	17	3 (1 + 2)
4 sites	175	23	6 (2 + 4)
5 sites	340	45	11 (4 + 7)
6 sites	347	46	14 (9 + 5)
7 sites	431	59	21 (16 + 5)
8 sites	476	65	28 (23 + 5)
9 sites	508	67	30 (26 + 4)
10 sites	534	72	32 (27 + 5)
11 sites	558	74	35 (32 + 3)
12 sites	577	77	39 (35 + 4)
13 sites	606	80	42 (37 + 5)
14 sites	661	86	44 (39 + 5)
15 sites	714	90	46 (41 + 5)
16 sites	818	103	53 (47 + 6)
17 sites	875	110	63 (59 + 4)
18 sites	913	114	67 (63 + 4)

Numbers marked in red in parentheses represent site-dominant components, and in black represent mixed components (related to both site differences and signal variables).

**FIGURE 2:**
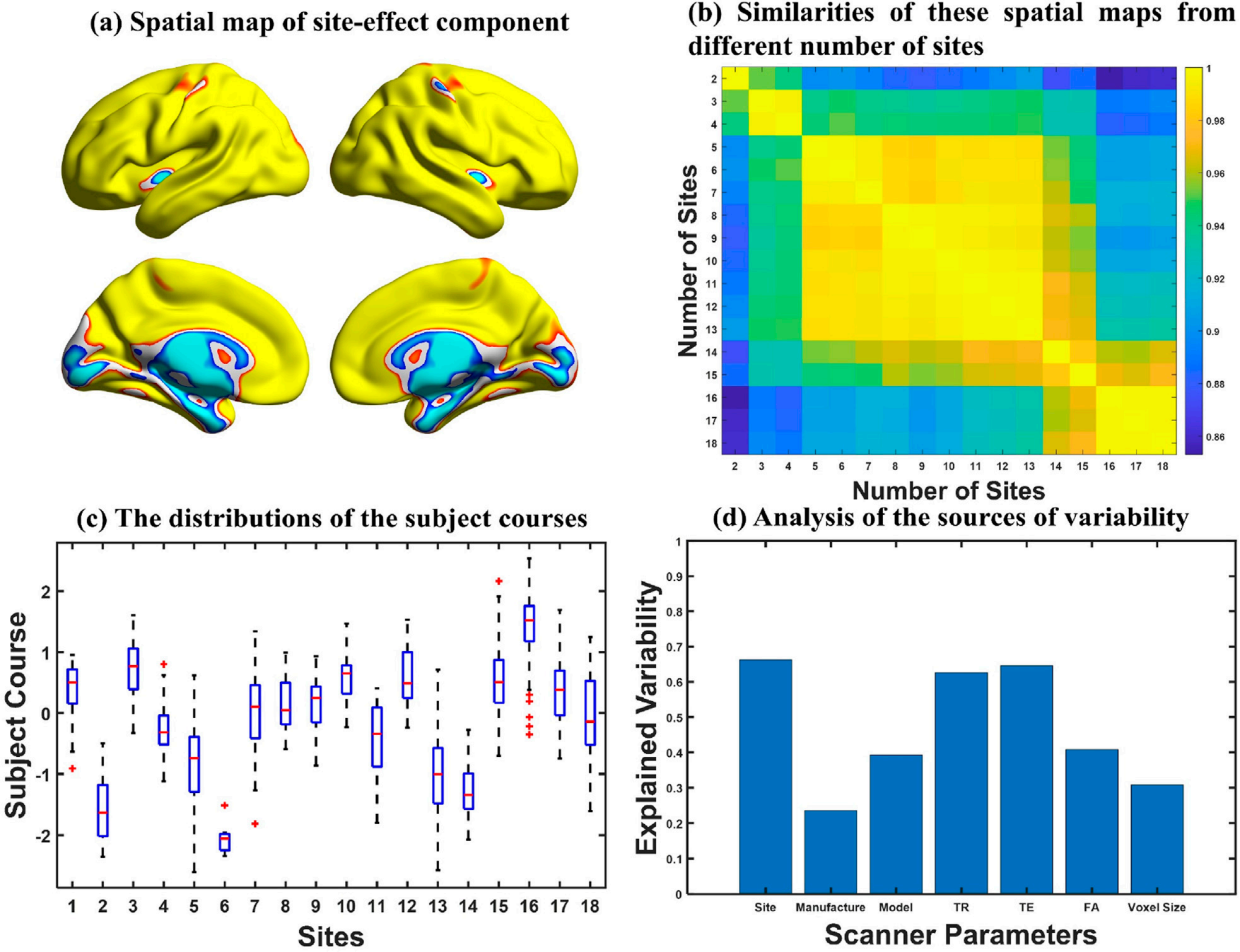
Representative LICA-derived global site-effect pattern in GM volume: IC1. **(a)** Spatial map of the component. **(b)** Spatial correlation coefficients, computed as the Pearson correlation in MATLAB, across different site-count subsets. **(c)** Distribution of subject loadings by site. **(d)** Relationship between loadings and acquisition parameters, quantified by LOOCV.

**FIGURE 3:**
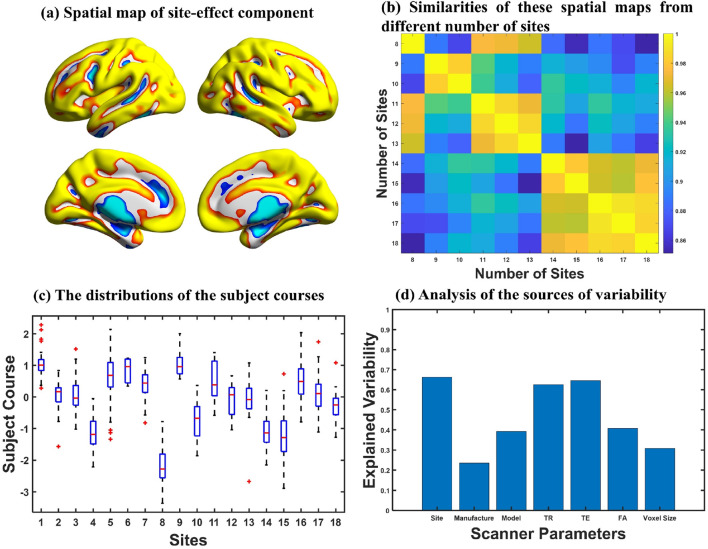
Representative LICA-derived global site-effect pattern in GM volume: IC2. **(a)** Spatial map of the component. **(b)** Spatial correlation coefficients, computed as the Pearson correlation in MATLAB, across different site-count subsets. **(c)** Distribution of subject loadings by site. **(d)** Relationship between loadings and acquisition parameters, quantified by LOOCV.

**FIGURE 4:**
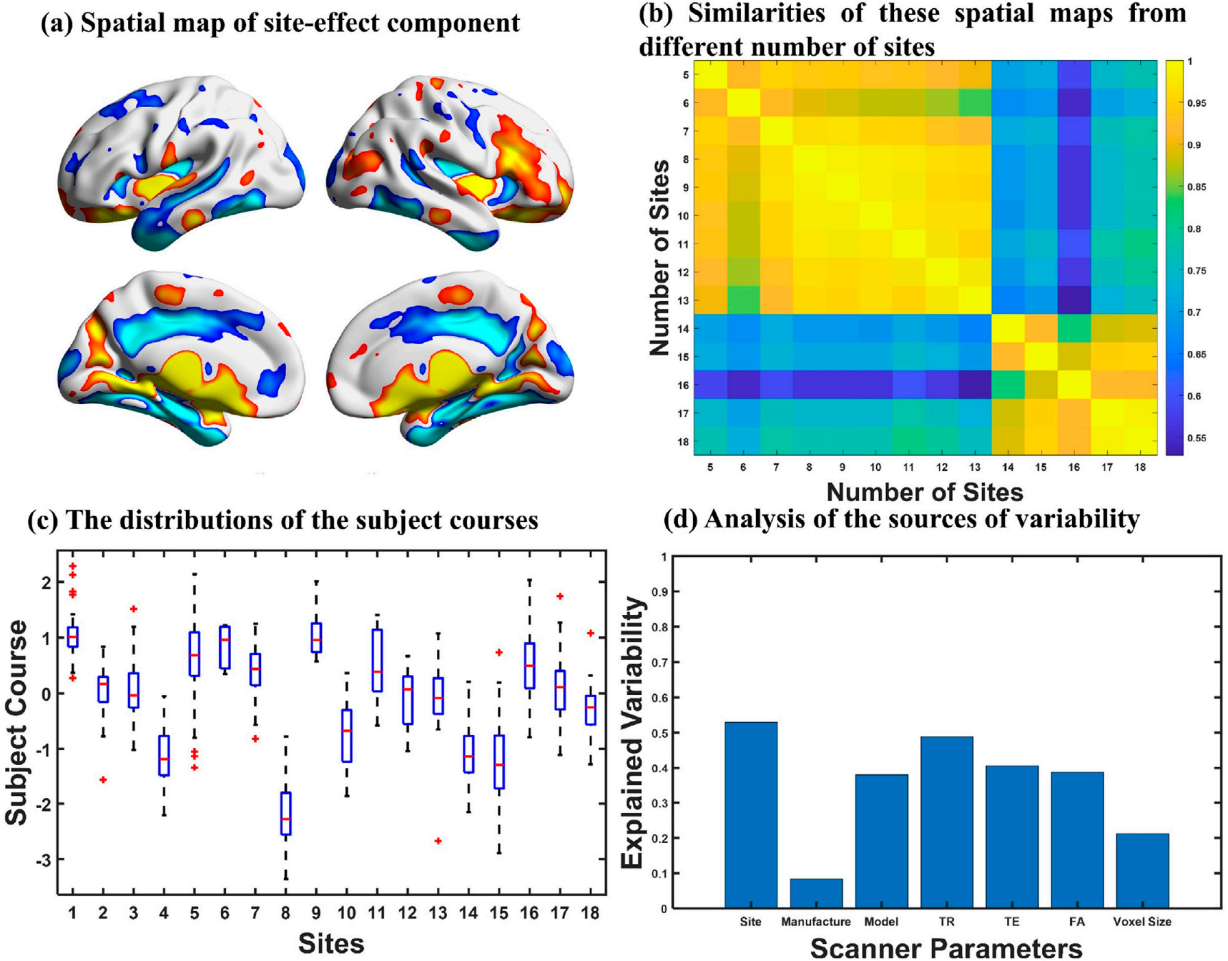
Representative LICA-derived site-effect pattern in GM volume: IC3. **(a)** Spatial map of the component. **(b)** Spatial correlation coefficients, computed as the Pearson correlation in MATLAB, across different site-count subsets. **(c)** Distribution of subject loadings by site. **(d)** Relationship between loadings and acquisition parameters, quantified by LOOCV.

The stepwise site-inclusion analysis showed that structural site-effect patterns emerged progressively as additional sites were incorporated. Rather than appearing as a single fixed pattern, site-related components varied in their detectability and spatial stability across different site compositions. Some components were already present when only a small number of sites were included, whereas others became detectable only after sites with additional scanner models or acquisition settings entered the analysis. These observations suggest that the structural site-effect patterns captured by LICA were sensitive to the increasing heterogeneity of scanner-protocol configurations, including differences in manufacturer, model, TR, TE, FA, and voxel size. Representative examples of these patterns are shown in Figures 2–4.

The representative GM components illustrated three forms of structural site variability: a highly stable global pattern (IC1), a broadly distributed site- or configuration-dependent pattern (IC2), and a localized regional pattern (IC3). Together, these results indicate that structural site effects were not limited to a uniform whole-brain shift, but included global, configuration-specific, and localized components.

Figure 2 shows the most representative structural site-effect pattern, IC1, which exhibited a widespread GM pattern across the brain. This component was highly reproducible in the stepwise site-inclusion analysis, it emerged with as few as two sites, and remained highly stable as the number of sites increased (Figure 2b), with similarity consistently exceeding 0.86. The corresponding subject loadings showed clear separation across acquisition sites, indicating that IC1 captured systematic between-site variability rather than random inter-subject variation. The parameter-explainability analysis showed that the site label explained the largest proportion of IC1 loading variance, a pattern consistently observed across subsequent representative components and therefore not repeated below. Among the recorded acquisition parameters, TR and TE accounted for the largest proportion of predictable variance in IC1 loadings, with $R_{\text{LOO}}^{2}$ ​ reaching approximately 0.7. These results indicate that the major site effects on GM images are widespread throughout the whole brain and remain highly stable across increasing numbers of sites.

IC2 showed another broadly distributed GM site-effect pattern, but its emergence was more strongly tied to site composition. This component became evident when eight sites were included and remained detectable in subsequent site-subset analyses. The distinct loading distribution associated with the site introduced at this stage suggests that IC2 captured a site-specific or scanner–protocol-specific source of structural variability, rather than a general effect shared equally across all sites. Thus, IC2 illustrates how the stepwise site-inclusion analysis can distinguish a stable global site-effect pattern from a component driven more strongly by a particular site or protocol configuration.

Figure 4 shows IC3, a structural site-effect pattern with a more localized GM configuration than IC1 and IC2. The spatial map included regionally specific clusters rather than a diffuse whole-brain distribution, with prominent involvement of the brainstem, posterior cingulate cortex, and temporal–occipital fusiform regions. This localized pattern suggests that structural site effects may affect specific anatomical regions rather than only producing diffuse whole-brain shifts. For downstream anatomical or clinical analyses, such localized site-effect patterns are important because they may overlap with regions of biological interest and therefore complicate interpretation if not recognized. In the stepwise site-inclusion analysis, IC3 first appeared when five sites were included and remained detectable in subsequent analyses up to the full 18-site dataset. Spatial correlations between the site-subset maps and the full-dataset reference map were generally high, although the correlation range was broader than that observed for IC1 and IC2, with values ranging from approximately 0.53–0.99. After site 14 was included, this effect exhibited two distinct distribution patterns across sites, suggesting that the observed site effect was primarily driven by individual sites and mainly affected localized GM regions. The subject-level loadings showed between-site differences with several sites exhibiting relatively distinct loading distributions. In the acquisition-parameter analysis, TR explained the largest proportion of predictable variance in IC3 loadings, with $R_{\text{LOO}}^{2}$ of approximately 0.5.

Together, the GM results indicate that structural site effects include at least three forms: a stable global component, a site-specific or configuration-specific component, and a localized regional component. This supports the need for component-level characterization, because a single site covariate or global correction term may not capture the spatial diversity of structural site effects.

### 3.2 Site-effect patterns in ALFF

For the ALFF analysis, voxel-wise ALFF maps from 795 participants across 16 sites were submitted to the same modality-wise LICA framework. Compared with the structural MRI data, the resting-state fMRI protocols showed pronounced variation in acquisition settings directly related to BOLD signal sampling, including TR, TE, FA, and voxel size. Across sites, TR ranged from subsecond acquisitions to 3,000 ms, TE varied from 15 to 45 ms, FA ranged from 60° to 90°, and voxel size differed substantially across protocols. These variations are particularly relevant for ALFF because this measure is derived from the amplitude of low-frequency BOLD fluctuations and may therefore be sensitive to differences in temporal sampling, signal-to-noise characteristics, and spatial resolution.

In the full ALFF dataset with 16 sites included in LICA, 99 independent components were identified, of which 64 were significantly associated with site. Among these site-related components, 55 were classified as site-dominant components and 9 as mixed components associated with both site and at least one biological covariate.

The representative ALFF site-effect patterns showed heterogeneous spatial configurations, including broad and relatively focal functional patterns. These components emerged at different stages of the site-inclusion analysis, indicating variable detectability across site compositions. In the parameter-weight analysis, the site label showed the largest overall contribution, while the leading recorded parameters varied across components and mainly included FA, TR, and voxel size.

**FIGURE 5:**
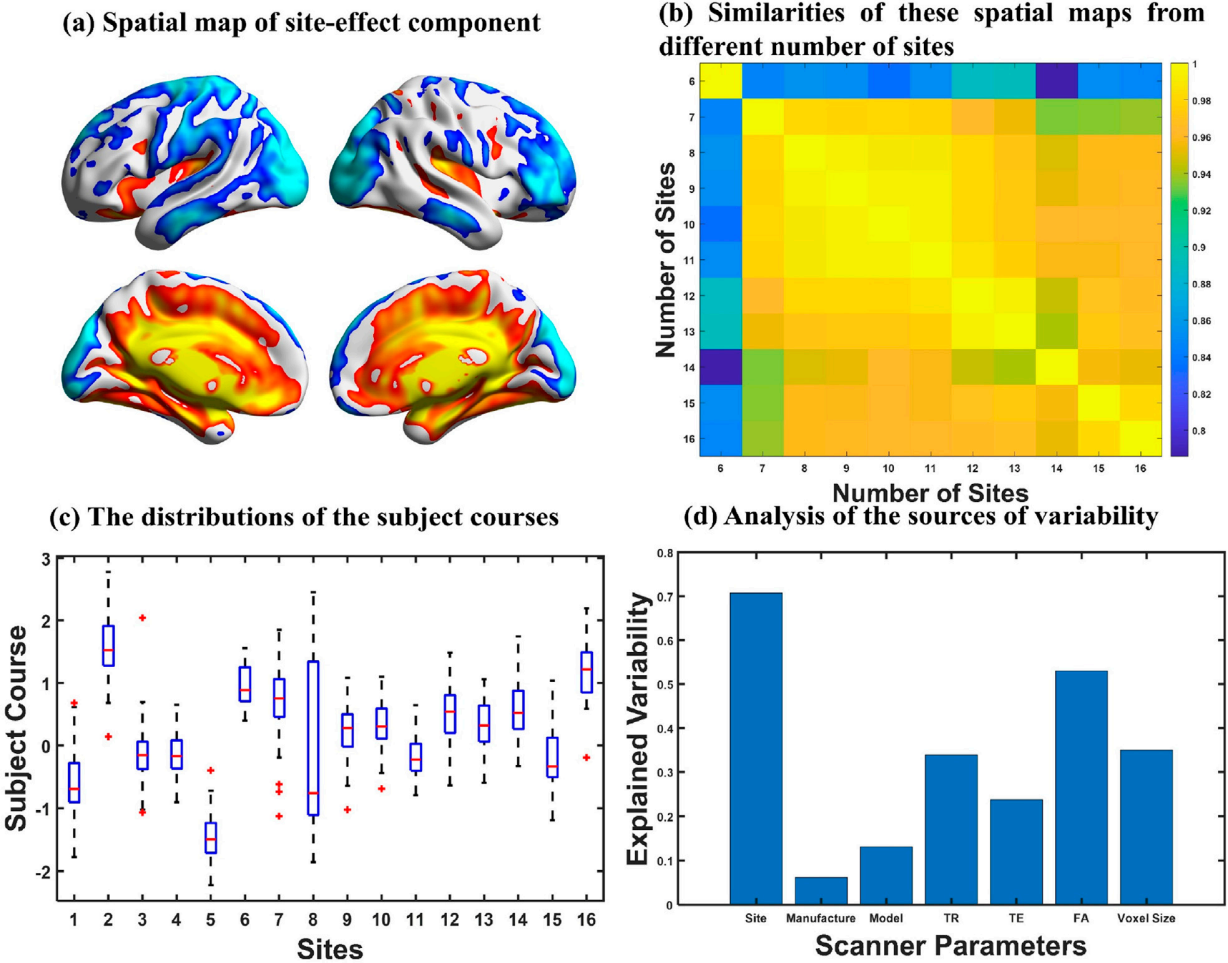
Representative LICA-derived global site-effect pattern in ALFF: IC1. **(a)** Spatial map of the component. **(b)** Spatial correlation coefficients, computed as the Pearson correlation in MATLAB, across different site-count subsets. **(c)** Distribution of subject loadings by site. **(d)** Relationship between loadings and acquisition parameters, quantified by LOOCV.

Figure 5 shows a representative ALFF site-effect pattern, IC1, with a broadly distributed spatial pattern in resting-state fluctuation amplitude. The component was detectable when five sites were included and showed consistent spatial correspondence with the full 16-site reference map, with spatial correlations ranging from 0.7857 to 0.9947. The subject-level loadings displayed clear between-site variation, indicating that the ALFF amplitude pattern differed systematically across acquisition sites. In the parameter-explainability analysis, FA accounted for the largest proportion of predictable variance in IC1 loadings, with $R_{\text{LOO}}^{2}$ of approximately 0.6.

**FIGURE 6:**
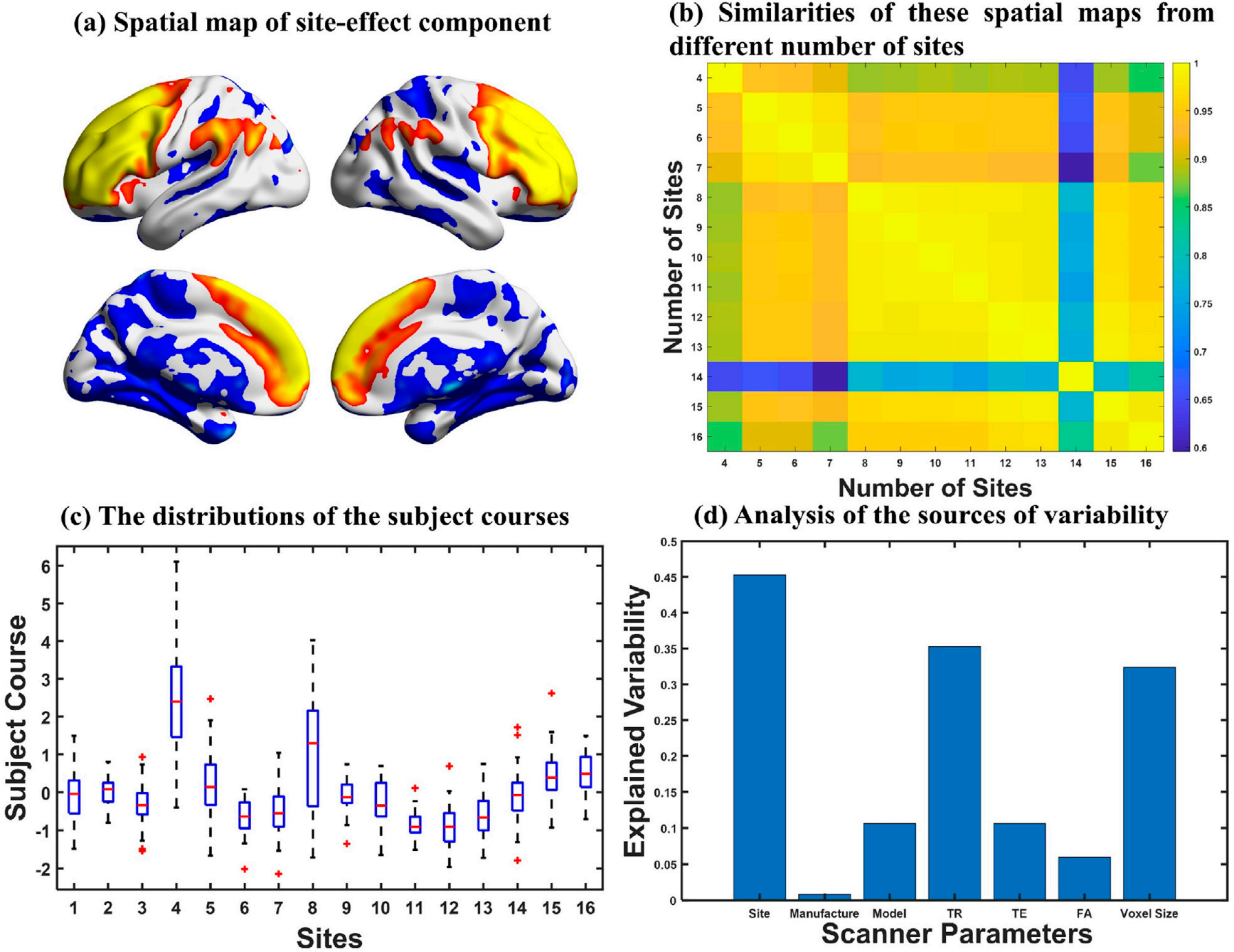
Representative LICA-derived global site-effect pattern in ALFF: IC2. **(a)** Spatial map of the component. **(b)** Spatial correlation coefficients, computed as the Pearson correlation in MATLAB, across different site-count subsets. **(c)** Distribution of subject loadings by site. **(d)** Relationship between loadings and acquisition parameters, quantified by LOOCV.

Figure 6 presents a second representative ALFF site-effect pattern, IC2. This component also displayed a near-global spatial pattern, with widespread ALFF variation across cortical and subcortical regions. In the stepwise site-inclusion analysis, IC2 first became evident when four sites were included and remained detectable in subsequent analyses. Spatial correlations between the site-subset maps and the full 16-site reference map ranged from 0.5957 to 0.9955. The subject-level loadings showed marked between-site differences, with several sites exhibiting shifted loading distributions relative to the others. In the acquisition-parameter analysis, TR and voxel size explained the largest proportion of predictable variance in IC2 loadings, with $R_{\text{LOO}}^{2}$ of approximately 0.35, suggesting that temporal sampling and spatial resolution contributed more strongly to this component. Therefore, although IC1 and IC2 were both spatially broad, they likely reflected different technical sources of functional site variability.

**FIGURE 7:**
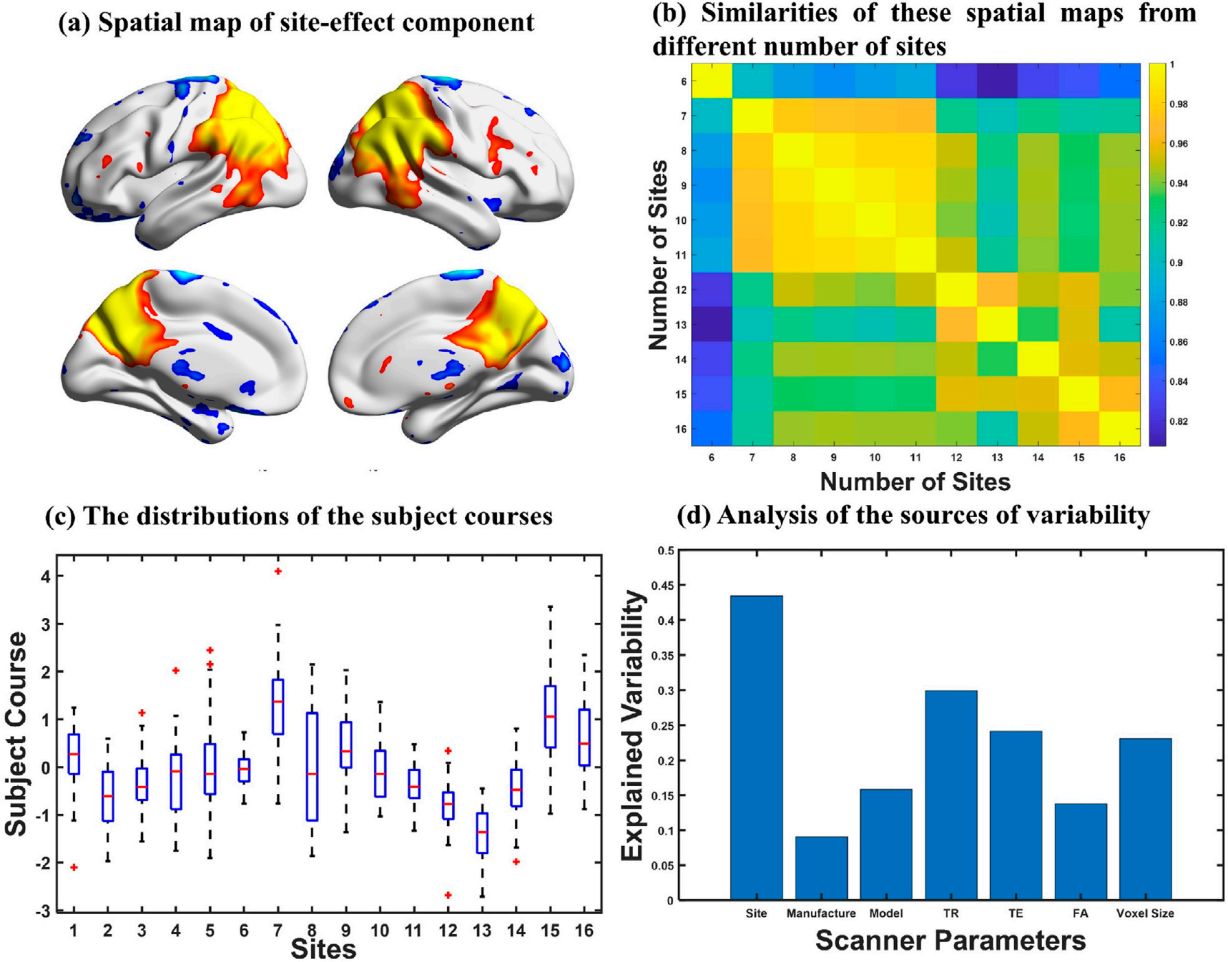
Representative LICA-derived site-effect pattern in ALFF: IC3. **(a)** Spatial map of the component. **(b)** Spatial correlation coefficients, computed as the Pearson correlation in MATLAB, across different site-count subsets. **(c)** Distribution of subject loadings by site. **(d)** Relationship between loadings and acquisition parameters, quantified by LOOCV.

Figure 7 shows IC3, an ALFF site-effect pattern with a more spatially restricted pattern than IC1 and IC2. The spatial map showed localized ALFF variation involving Frontal Pole and Superior Frontal Gyrus, rather than a near-global distribution. In the stepwise site-inclusion analysis, IC3 first became evident when six sites were included and remained detectable in subsequent analyses up to the full 16-site dataset. Spatial correlations between the site-subset maps and the full-dataset reference map ranged from 0.8071 to 0.9926. In the acquisition-parameter analysis, TR explained the largest proportion of predictable variance in IC3 loadings, with $R_{\text{LOO}}^{2}$ of approximately 0.3. These results indicate that functional site effects can also appear as localized patterns, not only as global BOLD-amplitude shifts. Such localized ALFF site-effect patterns may be particularly relevant for downstream studies focusing on region-specific functional activity.

Overall, the ALFF results indicate that functional site effects are spatially and technically heterogeneous. Unlike the GM results, where TR and TE were consistently prominent among representative components, ALFF site-effect patterns showed component-specific associations with FA, TR, and voxel size. This supports the view that site effects in resting-state amplitude measures arise from multiple acquisition dimensions and should be interpreted at the component level.

### 3.3 Site-effect patterns in ReHo

LICA was next applied to ReHo maps from the 795 participants with resting-state fMRI data. In the full 16-site ReHo dataset, LICA identified 83 independent components, including 55 site-related components. Among them, 51 were classified as site-dominant components and 4 as mixed components. Representative ReHo site-effect patterns are shown in Supplementary Figures S1–S3. The representative ReHo site-effect patterns showed broad bilateral, scattered, and spatially discontinuous patterns. Their spatial reproducibility and emergence differed across site-inclusion analyses. In the parameter-weight analysis, the site label remained the dominant explanatory factor, whereas FA, TR, voxel size, and scanner model showed component-dependent contributions.

Supplementary Figures S1 shows a representative ReHo site-effect pattern, IC1. The spatial map displayed a widespread pattern across cortical and subcortical regions, with both positive and negative ReHo loadings distributed bilaterally. In the site-inclusion analysis, this component was observed from the five-site analysis onward. Spatial correlations between the site-subset maps and the full 16-site reference map were generally high, with most values above approximately 0.80 and several site combinations approaching 1.0. The subject-level loadings showed clear between-site variation, with site 5 showing relatively high positive loadings and site 14 showing relatively low negative loadings compared with the other sites. In the acquisition-parameter analysis, the site label explained the largest proportion of variance in IC1 loadings. Among the recorded scanner parameters, FA showed the highest explanatory value, followed by TR, scanner model, voxel size, TE, and manufacturer.

Supplementary Figures S2 presents a second representative ReHo site-effect pattern. The spatial map showed a scattered ReHo pattern rather than a global distribution, with spatially discontinuous positive and negative clusters distributed across both hemispheres. In the site-inclusion analysis, IC2 was detected from the five-site analysis onward and remained present in subsequent site-subset analyses. The spatial similarity matrix showed relatively high correlations across most site combinations, with most values above approximately 0.85 after this component emerged. The subject-level loadings varied across sites, with relatively high positive loadings at sites 7 and 16 and lower loading distributions at sites 1, 4, and 10. In the acquisition-parameter analysis, the site label explained the largest proportion of variance in IC2 loadings. Among the recorded acquisition parameters, TR showed the highest explanatory value, followed by voxel size, TE and FA, scanner model, and manufacturer.

Supplementary Figures S3 shows ReHo IC3. The spatial map displayed a broad bilateral pattern. In the site-inclusion analysis, this component was observed from the three-site analysis onward. The spatial similarity matrix showed high correlations for many site-subset combinations, particularly among analyses including three to eleven sites and the full 16-site solution, while lower correlations were observed in several combinations involving twelve to fourteen sites. The subject-level loadings showed visible between-site differences, with relatively higher loading distributions at sites 4, 6, 9, and 10 and lower distributions at sites 12 and 14. In the acquisition-parameter analysis, the site label explained the largest proportion of variance in loadings. Among the recorded scanner parameters, scanner model and voxel size showed the highest explanatory values, followed by TE, whereas TR, FA, and manufacturer showed lower explanatory values.

Together, these ReHo results indicate that site effects in local functional synchronization were spatially heterogeneous and component-dependent, with different representative components showing distinct acquisition-parameter profiles.

## 4 Discussion

In this study, we developed a modality-wise LICA framework to identify, characterize, and interpret site-effect patterns in multi-site MRI data. Using structural GM volume and two resting-state functional measures, ALFF and ReHo, we showed that site-related variability was detectable across both structural and functional MRI measures. Rather than appearing as a uniform global offset, site effects were expressed as a spectrum of spatially organized patterns, including stable whole-brain, site-specific, regional, and scattered configurations. The stepwise site-inclusion analysis further showed that site-effect patterns were not fixed: some components emerged early and remained reproducible as more sites were added, whereas others became detectable only after specific scanner–protocol configurations entered the dataset, indicating a close relationship between site number, acquisition heterogeneity, and component detectability. Clear modality differences were also observed. GM volume showed a dominant and highly stable whole-brain site-effect pattern, together with additional site-specific or localized components, whereas ALFF and ReHo exhibited more heterogeneous functional patterns without a single dominant template. Parameter-weight analysis further indicated that site label made the largest overall contribution to component loadings, while recorded acquisition parameters showed modality-specific contributions: TR and TE were the main contributors to structural site effects, whereas FA, TR, voxel size, and scanner model contributed more prominently to functional site effects.

### 4.1 Site effects are expressed as spatially structured patterns

A central finding of this study is that site effects in multi-site MRI were expressed as spatially organized patterns rather than as a single global offset. In the GM analysis, the representative components showed different spatial extents: IC1 and IC2 were broadly distributed across the brain, whereas IC3 showed a more localized pattern involving the brainstem, posterior cingulate cortex, and temporal–occipital fusiform regions. This indicates that structural site effects can be expressed both as widespread GM variation and as regionally specific patterns.

A similar pattern-level organization was also observed in the functional measures. In ALFF, the representative components included near-global fluctuation-amplitude patterns as well as a more spatially restricted component, showing that site effects in low-frequency BOLD amplitude were not confined to a single spatial form. In ReHo, IC1 and IC3 showed broad bilateral patterns, whereas IC2 showed a more scattered and spatially discontinuous distribution. Thus, across GM volume, ALFF, and ReHo, site effects were expressed through multiple spatial configurations, including widespread, near-global, localized, and scattered patterns.

This spatial heterogeneity is important because different types of site-effect patterns may influence downstream analyses in different ways. Widespread site-effect patterns may affect global imaging summaries or multivariate models that aggregate information across large brain areas. More localized or scattered patterns may instead interfere with region-specific analyses, especially when the affected regions overlap with areas relevant to a clinical or biological hypothesis. The present results therefore support the need to examine site effects at the component and spatial-pattern level, rather than treating site variability only as a scalar nuisance term.

### 4.2 Site-effect patterns differ across structural and functional MRI measures

The modality-wise design allowed site-effect patterns to be examined separately in GM volume, ALFF, and ReHo. This was important because the three measures reflect different aspects of brain imaging data and are affected by different acquisition characteristics. GM volume is derived from T1-weighted anatomical contrast and spatial normalization, whereas ALFF and ReHo are derived from resting-state BOLD time series and therefore depend on temporal sampling, BOLD contrast, motion correction, and spatial resolution. The results showed that site-related components were present in all three imaging measures, but their spatial expression and parameter associations were not identical.

In GM volume, the representative site-effect patterns mainly reflected structural variation in GM maps. IC1 and IC2 showed broadly distributed spatial patterns, whereas IC3 showed a more localized distribution involving the brainstem, posterior cingulate cortex, and temporal–occipital fusiform regions. The parameter-explainability analysis also showed a relatively consistent pattern across the representative GM components: TR and TE explained the largest proportion of predictable variance for IC1 and IC2, while TR was the leading recorded parameter for IC3. This suggests that, in the structural MRI analysis, temporal acquisition parameters were closely associated with the identified GM site-effect patterns.

In ALFF, the representative components reflected site-related variation in low-frequency BOLD fluctuation amplitude rather than anatomical morphology. Compared with GM volume, the ALFF site-effect patterns were expressed in functional amplitude maps and showed different spatial configurations across components, including broad or near-global patterns and more spatially restricted patterns. The parameter-explainability profiles also differed from those observed in GM volume, with functional acquisition settings such as FA, voxel size, TR, and TE contributing to the predictable variance of component loadings. These results indicate that ALFF site effects were not a direct functional counterpart of the GM site effects, but reflected modality-specific variability in resting-state BOLD amplitude.

In ReHo, the site-effect patterns were again different from both GM volume and ALFF. ReHo IC1 showed a widespread pattern across cortical and subcortical regions, IC2 showed a scattered and spatially discontinuous pattern, and IC3 showed a broad bilateral distribution. The acquisition-parameter profiles were also component-dependent: FA showed the highest explanatory value among recorded parameters for ReHo IC1, TR was the leading parameter for ReHo IC2, and scanner model together with voxel size showed the highest explanatory values for ReHo IC3. Thus, even within resting-state fMRI, ALFF and ReHo showed different site-effect expressions, consistent with the fact that ALFF measures fluctuation amplitude whereas ReHo measures local temporal coherence.

Together, these modality-specific findings show that site effects cannot be assumed to have a single spatial or technical form across MRI measures. A parameter that is strongly associated with one site-effect pattern or one modality may have weaker explanatory value for another. Therefore, analyzing GM volume, ALFF, and ReHo separately was useful for distinguishing structural and functional expressions of site-related variability, while avoiding the ambiguity that could arise if the measures were fused before the site-effect patterns were characterized.

### 4.3 Recorded acquisition parameters explain part, but not all, site-related variance

The parameter-explainability analysis showed that recorded acquisition parameters were associated with the loadings of site-related components, but their explanatory power was generally weaker than that of the site label itself. This pattern was observed across structural and functional MRI measures. In GM volume, temporal acquisition parameters such as TR and TE contributed to the representative site-effect patterns. In ALFF and ReHo, parameters related to BOLD acquisition and spatial sampling, including FA, voxel size, TR, TE, and scanner model, showed component-dependent contributions. However, no single recorded parameter consistently accounted for the full site-related variance across components or modalities.

This result is important for interpreting site effects in multi-site MRI. A site label is not equivalent to one acquisition parameter. Instead, it represents a composite scanner–protocol environment that may include scanner manufacturer and model, head coil, software version, pulse-sequence implementation, reconstruction procedure, local protocol choices, operator-related factors, and site-specific quality-control procedures. Many of these factors are either unavailable in public datasets or difficult to encode as explicit covariates. Therefore, the stronger explanatory role of the site label indicates that site effects reflect a combination of measured and unmeasured technical factors, rather than the influence of a single recorded parameter.

The observed difference between site-level and parameter-level explainability also supports the need for component-level analysis. If only site labels are modeled, the analysis can detect the presence of inter-site variability but cannot indicate which acquisition factors may contribute to it. Conversely, if only individual scanner parameters are examined, a substantial portion of site-related variance may remain unexplained because the recorded metadata do not fully describe the acquisition environment. By combining component-wise site association with parameter-level $R_{\text{LOO}}^{2}$ , the proposed framework provides a way to distinguish broad site-level effects from the partial contributions of measurable scanner parameters. Importantly, the contribution of acquisition parameters should be interpreted cautiously. In ABIDE II, scanner and protocol parameters were not independently manipulated, and parameters such as scanner model, TR, TE, FA, and voxel size often varied together within site-specific acquisition settings. Therefore, the present analysis cannot isolate the independent causal effect of each parameter or quantify their interaction effects in a controlled-variable manner. The parameter-explainability results should instead be viewed as indicators of how strongly recorded scanner and protocol variables predict component-loading variability under the available multi-site data structure.

### 4.4 Component emergence and site-wise loading distributions reveal different forms of site effects

The stepwise site-inclusion analysis provided information that could not be obtained from the full-dataset decomposition alone. In the full dataset, a component can be identified as site-related if its loadings differ significantly across sites. However, this result does not indicate whether the component reflects a site effect driven mainly by one newly introduced scanner–protocol configuration, or a more distributed site effect involving gradual differences across multiple sites. By repeating LICA after progressively adding sites, the present framework allowed us to examine when each site-effect pattern first appeared and how its subject-level loadings were distributed across sites.

One type of site-effect pattern was closely linked to the introduction of a particular site. GM IC2 provides such an example. This component was not observed in the earliest site-subset analyses, but became detectable when eight sites were included and remained stable in subsequent analyses. Its loading distribution also showed a clear shift for the site introduced at this stage, separating it from most other sites. This pattern suggests that the component was not a general site effect shared across all sites, but was strongly driven by one site or by a small subset of sites with distinctive scanner–protocol settings. In this case, the spatial component may reflect a site-specific or configuration-specific source of acquisition heterogeneity.

A second type was represented by distributed site-effect patterns, in which no single site formed an isolated loading cluster. For these components, the loadings varied across many sites, with different sites showing gradual shifts or partially overlapping distributions. This pattern is less consistent with a single-site outlier effect and more consistent with cumulative inter-site variability arising from differences in scanner manufacturer, scanner model, sequence parameters, voxel size, and other protocol-related factors. In such cases, the component reflects a broader multi-site measurement pattern rather than the effect of one newly introduced site alone.

This distinction between site-specific and distributed site-effect patterns is important for interpreting LICA-derived site-related components. A component that appears only after a particular site is introduced and shows a marked loading shift for that site may reflect a site-specific or protocol-specific effect. In contrast, a component that is reproducibly detected across site compositions and shows graded loading differences across multiple sites is more consistent with distributed inter-site heterogeneity rather than a single-site driver.

### 4.5 Relationship to existing harmonization approaches

The proposed framework differs from conventional multi-site harmonization methods in its primary objective. Methods such as ComBat, GLM-based adjustment, ICA-based denoising, and deep-learning harmonization are mainly designed to reduce or remove site-related variability so that downstream analyses can be performed on pooled multi-site data. In contrast, the present framework aims to characterize site effects themselves before removal. Specifically, it identifies spatially organized site-effect patterns, evaluates their reproducibility across different site compositions, and links component loadings to recorded scanner and acquisition parameters.

This distinction is important because removal and interpretation address different methodological questions. ComBat and related statistical harmonization methods can reduce distributional differences across sites, but they usually do not indicate where site effects are spatially expressed or whether the corrected variance corresponds to a stable scanner–protocol pattern. ICA-based denoising methods can identify structured components related to scanner or site effects, but they are often used primarily for denoising rather than for systematic stability and parameter-explainability analysis. Deep-learning harmonization methods provide flexible nonlinear transformations, but the resulting corrections are often less directly interpretable in terms of spatial brain patterns and acquisition parameters. The proposed LICA-based framework therefore provides complementary information: it does not replace harmonization, but offers a component-level diagnostic analysis that can inform which site-effect patterns are stable, which are site-specific, which are mixed with biological variables, and which acquisition factors may contribute to them.

### 4.6 Practical implications for downstream analysis, harmonization, and biomarker studies

The proposed framework has practical implications for multi-site MRI studies because it provides diagnostic information before downstream statistical analysis or harmonization is performed. Rather than treating site effects as a single nuisance term, the framework identifies spatially organized site-effect patterns, evaluates their stability across site compositions, and quantifies their association with recorded scanner and acquisition parameters. These outputs can help researchers determine whether site-related variability is widespread, localized, site-specific, stable across site subsets, or mixed with biological variables.

For downstream group-comparison or prediction analyses, the identified site-effect patterns can be used as a quality-control and interpretive reference. If a candidate disease-related effect or biomarker overlaps strongly with a stable site-effect pattern, the result should be interpreted cautiously because it may be vulnerable to scanner- or protocol-related variability. Conversely, effects located outside the dominant site-effect patterns may be less directly affected by the main sources of site variability identified in the dataset. In this sense, the framework can help evaluate the robustness of downstream findings and distinguish biologically meaningful effects from patterns that may be strongly influenced by site-specific measurement conditions.

For harmonization strategies, the component classification provides information that may guide how site-related variance should be handled. Site-dominant components may be candidates for removal or stronger adjustment, whereas mixed components require more cautious treatment because they are associated with both site labels and biological variables. Removing such mixed components without additional consideration may reduce site effects but may also attenuate biologically relevant variation. Therefore, the proposed framework can support more selective and interpretable harmonization strategies, rather than applying uniform correction to all site-associated variance.

For biomarker studies, the framework can be used to assess whether candidate imaging biomarkers are spatially or statistically entangled with site-effect patterns. This is particularly relevant in retrospective multi-site datasets, where diagnostic group, age, sex, scanner hardware, and acquisition protocol may vary together across sites. By identifying stable site-effect patterns and their acquisition-parameter associations, the framework provides an additional layer of evidence for judging whether a candidate biomarker is likely to be robust across sites or sensitive to site-specific acquisition conditions.

Finally, the acquisition-parameter explainability analysis may inform future study design. Parameters that consistently explain the loadings of stable site-effect patterns can indicate which aspects of scanner or protocol variation deserve closer control in prospective multi-site data collection. Although the present study does not directly test downstream biomarker performance after harmonization, it provides a diagnostic basis for designing more targeted harmonization, quality-control, and protocol-standardization strategies in future multi-site MRI studies.

### 4.7 Limitations

Several limitations should be considered. First, this study was based on observational multi-site MRI data, in which scanner hardware, software environment, acquisition protocols, and participant characteristics were not independently manipulated. Many acquisition parameters were partially coupled with site labels; for example, scanner model, TR, TE, FA, and voxel size often varied together within the same site-specific protocol. Therefore, although the proposed framework quantified the extent to which recorded acquisition parameters explained component loadings, these associations should not be interpreted as strict causal effects of individual parameters. The parameter-explainability analysis instead provides an interpretable estimate of how strongly recorded technical factors are associated with each site-effect pattern under the available acquisition structure.

Second, the present study did not include traveling-subject or phantom data. As a result, we could not fully separate measurement bias, which reflects scanner- or protocol-induced differences in image formation, from sampling bias, which reflects differences in participant composition across sites. This distinction is particularly important in multi-site neuroimaging because site labels may capture both technical measurement variability and site-specific demographic or clinical structure. Although diagnosis, age, and sex were included as biological covariates in the component-classification and parameter-explainability analyses, unmeasured participant-level or protocol-level factors may still contribute to the identified site-effect patterns. Future studies incorporating traveling-subject designs, phantom scans, or prospectively harmonized acquisition protocols would provide stronger evidence for disentangling measurement-related site effects from sampling-related variability. Although the effects of measured biological variables were regressed out from mixed-component loadings before acquisition-parameter interpretation, this adjustment cannot fully eliminate residual confounding. In ABIDE II, diagnostic status, demographic characteristics, scanner hardware, and acquisition protocols may still vary together across sites. Therefore, the acquisition-parameter explainability results should be interpreted as reflecting the contribution of recorded scanner and protocol variables to residual component-loading variability under the available dataset structure.

Third, we did not perform an exhaustive sensitivity analysis across multiple preset LICA dimensionalities. Although LICA uses a Bayesian estimation framework that can down-weight weak components, the preset model order may still influence component separation, particularly in joint multimodal fusion settings. In the present modality-wise analysis, we therefore used a conservative high-dimensional initialization and focused the robustness assessment on spatial reproducibility across different site compositions. Future studies could systematically examine how alternative dimensionality settings affect the detection and interpretation of site-related components and their site-effect patterns.

## 5 Conclusion

In this study, we developed a LICA-based framework for identifying, characterizing, and interpreting site-effect patterns in multi-site MRI data. Applying this framework separately to GM volume, ALFF, and ReHo maps from ABIDE II showed that site-related variability was present across both structural and resting-state functional MRI measures. The identified components were not limited to uniform global shifts, but appeared as spatially structured patterns with different anatomical extents, including widespread, localized, and scattered distributions.

By combining component-wise association analysis, stepwise site-inclusion stability assessment, and $R_{\text{LOO}}^{2}$ -based parameter explainability, the proposed framework provided a systematic way to evaluate site effects beyond simple nuisance regression. The stability analysis showed that some site-effect patterns were reproducible across multiple site compositions, whereas others emerged only after specific scanner–protocol configurations were introduced. The parameter-explainability analysis further showed that recorded acquisition parameters, including TR, TE, FA, voxel size, and scanner model, explained part of the component-level site variability, but their explanatory power was generally weaker than that of the site label itself. This indicates that site acts as a composite proxy for both recorded and unrecorded technical factors.

Overall, these findings support a shift from merely removing site effects toward mapping, stabilizing, and technically interpreting them. The proposed framework can serve as a component-level diagnostic tool for multi-site neuroimaging studies, helping to distinguish site-dominant components from mixed components, evaluate the reproducibility of site-effect patterns, and identify acquisition factors that contribute to inter-site variability. This information may guide downstream statistical interpretation, support more selective harmonization strategies, and help assess whether candidate imaging biomarkers are vulnerable to site-related spatial patterns. Such information may improve the interpretability, reproducibility, and methodological transparency of future multi-site structural and functional MRI analyses.

## Data Availability

Publicly available datasets were analyzed in this study. These data can be found here: ABIDE II data are publicly available at (http://fcon_1000.projects.nitrc.org/indi/abide/abide_II.html).
