# Supplementary material for: A linked independent component analysis framework for characterizing site-effect patterns in multi-site structural and functional MRI
Source: Front Bioinform. 2026 Jul 29;6:1902380. doi: 10.3389/fbinf.2026.1902380 (PMC13461629; doi:10.3389/fbinf.2026.1902380)
Supplement: Supplementary file 1 [file Supplementaryfile1.docx]

Supplementary Material

# 1 Supplementary Figures and Tables

## 1.1 Supplementary Figures

**Supplementary Figure 1.**

Representative LICA-derived site-effect pattern in ReHo: IC1. (a) Spatial map of the component. (b) Spatial correlation coefficients, computed as the Pearson correlation in MATLAB, across different site-count subsets. (c) Distribution of subject loadings by site. (d) Relationship between loadings and acquisition parameters, quantified by LOOCV

**Supplementary Figure 2.**

Representative LICA-derived site-effect pattern in ReHo: IC2. (a) Spatial map of the component. (b) Spatial correlation coefficients, computed as the Pearson correlation in MATLAB, across different site-count subsets. (c) Distribution of subject loadings by site. (d) Relationship between loadings and acquisition parameters, quantified by LOOCV

**Supplementary Figure 3.**

Representative LICA-derived site-effect pattern in ReHo: IC3. (a) Spatial map of the component. (b) Spatial correlation coefficients, computed as the Pearson correlation in MATLAB, across different site-count subsets. (c) Distribution of subject loadings by site. (d) Relationship between loadings and acquisition parameters, quantified by LOOCV
